# Selective Upregulation by Theanine of Slc38a1 Expression in Neural Stem Cell for Brain Wellness

**DOI:** 10.3390/molecules25020347

**Published:** 2020-01-15

**Authors:** Yukio Yoneda, Koichi Kawada, Nobuyuki Kuramoto

**Affiliations:** 1 Department of Pharmacology, Osaka University Graduate School of Dentistry, Suita 565-0871, Japan; 2 The Institute of Prophylactic Pharmacology, Kita-Shinagawa, Shinagawa, Tokyo 140-0001, Japan; kkawada@cis.ac.jp (K.K.); kuramoto@pharm.setsunan.ac.jp (N.K.); 3 Department of Pharmacology, Chiba Institute of Science Faculty of Pharmaceutical Sciences, Chiba 288-0025, Japan; 4 Laboratory of Molecular Pharmacology, Setsunan University Faculty of Pharmaceutical Sciences, Hirakata 573-0101, Japan

**Keywords:** theanine, glutamine transporter, Slc38a1, neural stem cell, mTOR, neurogenesis

## Abstract

Theanine is an amino acid abundant in green tea with an amide moiety analogous to glutamine (GLN) rather than glutamic acid (Glu) and GABA, which are both well-known as amino acid neurotransmitters in the brain. Theanine has no polyphenol and flavonoid structures required for an anti-oxidative property as seen with catechins and tannins, which are more enriched in green tea. We have shown marked inhibition by this exogenous amino acid theanine of the uptake of [^3^H]GLN, but not of [^3^H]Glu, in rat brain synaptosomes. Beside a ubiquitous role as an endogenous amino acid, GLN has been believed to be a main precursor for the neurotransmitter Glu sequestered in a neurotransmitter pool at glutamatergic neurons in the brain. The GLN transporter solute carrier 38a1 (Slc38a1) plays a crucial role in the incorporation of extracellular GLN for the intracellular conversion to Glu by glutaminase and subsequent sequestration at synaptic vesicles in neurons. However, Slc38a1 is also expressed by undifferentiated neural progenitor cells (NPCs) not featuring a neuronal phenotype. NPCs are derived from a primitive stem cell endowed to proliferate for self-renewal and to commit differentiation to several daughter cell lineages such as neurons, astrocytes, and oligodendrocytes. In vitro culture with theanine leads to the marked promotion of the generation of new neurons together with selective upregulation of Slc38a1 transcript expression in NPCs. In this review, we will refer to a possible novel neurogenic role of theanine for brain wellness through a molecular mechanism relevant to facilitated neurogenesis with a focus on Slc38a1 expressed by undifferentiated NPCs on the basis of our accumulating findings to date.

## 1. Introduction

The prevailing view is that glutamic acid (Glu) plays a dual role in the mammalian central nervous system as an excitotoxic neurotoxin and as an excitatory neurotransmitter through a variety of ionotropic and metabotropic Glu receptor subtypes, besides a ubiquitous role as an energy source and a protein constituent in most eukaryotic cells. Activation of the *N*-methyl-d-aspartate (NMDA) subtype of ionotropic Glu receptors is required for the acquisition of neuronal plasticity such as learning and memory, whereas overactivation of the NMDA receptor subtype leads to neuronal cell death for neurodegeneration in the brain (for example, see review [1]). In contrast, the inhibitory neurotransmitter γ-aminobutyric acid (GABA) is synthesized from Glu not sequestered in synaptic vesicles by the catalytic action of Glu decarboxylase at GABAergic neurons in the brain.

The tea plant “Chanoki” (*Camellia sinensis*) contains theanine (l-γ-glutamylethylamide) as the major amino acid constituent besides several abundant ingredients such as caffeine and catechins. In the tea plant, theanine is made from both Glu and ethylamine through a mechanism related to the nitrogen supply absorbed from the roots for subsequent translocation of theanine to the leaves [2]. Theanine was first identified as an amide constituent of green tea in 1949 [3], and was shown to be enriched in high-quality green teas, such as Gyokuro and Matcha, rather than normal-grade green teas [4]. Theanine is also found in both black and oolong teas, which are similarly made from the identical tea plant with different manufacturing processes [5].

On the other hand, evidence is accumulating for the beneficial effects of theanine for alleviating different brain dysfunctions in in vitro and in vivo experiments. For instance, theanine prevented the aforementioned neurotoxicity mediated by overactivation of the NMDA receptor subtype in a cell model of Alzheimer’s disease in vitro [6], in spite of a considerably low affinity as an effective antagonist at any ionotropic subtypes of Glu receptors [7] as described below. Oral intake of theanine at 5–6 mg/kg improved learning impairment, shortened lifespan, cerebral atrophy, behavioral depression, and oxidative DNA damage in mice given sustained social stress in vivo [8]. We have shown that daily oral administration of theanine at 50 mg/kg for five days ameliorates behavioral abnormalities such as immobilization and hyper-locomotion seen in mice with prior severe traumatic stress, in addition to preventing the robust decline of the incorporation of 5-bromo-2′-deoxyuridine (BrdU), a thymidine analog, in the hippocampal dentate gyrus in vivo [9]. In our recent study using mice defective of klotho identified as an aging suppressor, marked alleviation was seen in memory impairment after oral intake of theanine at 4 mg/kg in drinking water for 24 days [10]. The chemical structure of theanine is closer to that of glutamine (GLN) rather than Glu and GABA with respect to the absence of a free carboxylic acid from the gamma carbon position [11] The structural analysis thus argues in favor of an idea that theanine could modulate a variety of functionalities of the endogenous GLN in the brain, including a canonical role as a precursor of the neurotransmitter Glu at glutamatergic neurons.

## 2. Theanine on Neural Stem Cells (NSCs)

### 2.1. Neural Progenitor Cells (NPCs)

In the brain, neurons are derived from neural stem cells (NSCs) defined as a primitive cell endowed to self-renewal by proliferation, as well as to commit differentiation to several daughter cell lineages, such as neuron, astrocyte, and oligodendrocyte [12,13,14,15]. By contrast, neural progenitor cells (NPCs) are often used experimentally as a mixture of the individual progenitor cells for neurons, astrocytes, and oligodendrocytes besides NSCs. During the in vitro culture in the presence of several growth factors of NPCs prepared from neocortex of embryonic rats [16] and mice [17], in addition to adult mouse hippocampus [18,19], round spheres named neurospheres are invariably formed by clustered dividing cells along with an increasing size proportional to the days in culture. These neurospheres are enriched of cells immunoreactive for a progenitor marker protein, but not for either a neuronal marker or an astrocytic marker. Upon subsequent culture of dispersed neurospheres in the absence of growth factors under adherent conditions, in contrast, cells become immunoreactive for either a neuronal marker protein or an astrocytic marker protein, but not for a progenitor marker protein. These cellular profiles give strong support for the validity of preparations experimentally isolated from embryonic and adult rodent brains as NPCs before commitment. The neurosphere size is adequately increasing in proportion to the days in culture as an index of the proliferative activity, while the differentiation activity is quantitatively estimated by counting the number of cells immunoreactive for each marker protein over the total number of cells in culture.

### 2.2. Modulation by Glu of Neurogenesis

In undifferentiated NPCs isolated from fetal rat and mouse neocortex, functional expression was seen for a variety of signaling machineries required for signal input at glutamatergic and GABAergic synapses in spite of the absence of any neuronal features [20]. For example, each transcript was markedly expressed for GluN1 and GluN2A-D subunits of NMDA receptors, GluA1-4 subunits of DL-α-amino-3-hydroxy-5-methylisoxasole-4-propionate receptors, and GluK1-2 subunits of kainite receptors in undifferentiated NPCs from embryonic rat neocortex, respectively [20]. Exposure to NMDA led to an increased level of intracellular free Ca^2+^ in an antagonist-sensitive manner in these NPCs before commitment. Glutamatergic signals are usually mediated by certain ionotropic and metabotropic receptor subtypes expressed at the cellular surface in neurons, astrocytes, and oligodendrocytes as described above. Activation of an ionotropic NMDA receptor subtype led to the suppression of proliferation activity along with promoted neuronal commitment activity in undifferentiated NPCs. However, a metabotropic group III Glu receptor subtype was responsible for the promotion of proliferation with a concomitant deterioration of neuronal commitment in NPCs before commitment. It thus appears that glutamatergic receptors required for signal input are already expressed functionally to modulate the activities for self-renewal and subsequent commitment to differentiate to particular daughter cell lineages even in undifferentiated NSCs, which have no clear phenotype of individual progeny cell lineages at all. Expression profiles of Glu receptor subtypes would be thus a determinant of future fates of primitive NSCs for the future adequate orchestration and harmonization of sound neuronal network in the adult brain.

### 2.3. Activation by Theanine of Embryonic Neurogenesis

On analogy to the aforementioned modulation by Glu, we have proceeded with studies on the possible role of the green tea amino acid theanine in the primitive features of NPCs in vitro. The exogenous amino acid theanine, but not the endogenous amino acid GLN, markedly increased the size of neurospheres, as well as the activity of 3-(4,5-dimethyl-2-thiazolyl)-2,5-diphenyl-2H-tetrazolium bromide (MTT) reduction, as an index of the proliferative activity in NPCs from embryonic rat neocortex [21]. In embryonic mouse NPCs [22], similarly, theanine markedly facilitated the incorporation of BrdU in a concentration-dependent manner, in addition to increasing the neurosphere size and MTT reduction. Furthermore, a marked promotion was invariably seen in spontaneous and induced commitment to differentiate into a neuronal lineage with attenuated astrocytic commitment. In addition to our in vitro studies mentioned above, theanine was shown to accelerate BrdU accumulation in the hippocampal dentate granule cell layer along with promoted object recognition memory in newborn rats chronically given oral theanine during pregnancy in vivo [23]. Theanine could thus promote both proliferation and neuronal commitment activities responsible for facilitated neurogenesis in a unique manner different from that by Glu. Glutamatergic signal inputs never simultaneously promote the proliferation and neuronal commitment activities in embryonic NPCs as described above.

### 2.4. Theanine on Adult Neurogenesis

Postnatal hippocampal neurogenesis was first reported in young adult rats in 1965 [24]. Since NSCs are shown to be enriched in particular restricted regions such as the subventricular zone (SVZ) and the subgranular zone (SGZ) of the hippocampal dentate gyrus in adult rodent brains [25], we have isolated the hippocampus from young adult mice with the overexpression of green fluorescent protein (GFP) in cells expressing the progenitor cell marker, nestin [26]. In fact, GFP fluorescence was predominantly found in the SGZ below the granular cell layer in hippocampal sections dissected from these nestin-GFP transgenic mice [22]. Prolonged culture with theanine significantly increased the size of neurospheres formed during the culture of hippocampal NPCs prepared from adult Nestin-GFP transgenic mice. Taken together, theanine would promote proliferation for self-replication during adult neurogenesis after chronic exposure.

Nevertheless, the significance of adult neurogenesis is still under debate with less quantitative analysis in human brains, in contrast to studies on experimental mammals. In adult human SVZ and SGZ, microglia were thought to be the majority of proliferating cells [27,28]. In the autopsied hippocampus of healthy human subjects at the age of 14 to 79 years old, by contrast, no marked difference was seen in the numbers of intermediate NPCs, immature neurons, and mature granule neurons across ages with preserved neurogenesis [29]. In recent reports, hippocampal neurogenesis was invariably seen in neurologically healthy human adults with a sharp decline in patients with Alzheimer’s disease [30]. Hippocampus was also shown to undergo neurogenesis even in aged adults and Alzheimer’s patients [31]. One of the reasons for these conflicting results could lie on variable postmortem times before fixation of sections from adult human brains. Postmortem time is supposed to be a determinant of the lifetime of vital NSCs expressed at particularly restricted regions in adult human brains, while we are unable to maintain consistency in the postmortem time for human specimens in contrast to experimental animal studies.

Moreover, we need to pay much attention to the species difference between rodents and primates with respect to adult neurogenesis. In contrast to rodent brains, for instance, human brains were enriched with striatal neurogenesis in place of olfactory bulb neurogenesis [29,32]. We thus have no choice but to wait for future innovative techniques to monitor in situ features of NSCs/NPCs expressed at particular niche regions in human brains for elucidating the physiological and pathological significance of adult neurogenesis, in place of the use of postmortem human specimens in current studies. As long as we deal with postmortem specimens for the analysis of adult neurogenesis in human brains, we are unable to reach a consistent conclusion on adult neurogenesis in human brains. The possibility that the controversial results are brought about by the variations of postmortem times up to fixation is not ruled out.

## 3. Correlation between Theanine and GLN Transporters

### 3.1. Selective Upregulation by Theanine of Slc38a1 Expression in NPCs

In undifferentiated NPCs isolated from embryonic rat [21] and mouse [22] neocortex, more than doubled upregulation was invariably found in transcript expression of the GLN transporter (GLNT) isoform, solute carrier 38a1 (Slc38a1), which is also referred to as a sodium-dependent neutral amino acid transporter-1 (SNAT1), after sustained exposure to theanine. However, theanine was negative in affecting the transcript expression of different trophic and adhesion factors, which are all shown to be capable of modulating the primitive properties of NSCs/NPCs in the literature. These included β-1 integrin, ciliary neurotrophic factor, ciliary neurotrophic factor receptor alpha, epidermal growth factor, N-cadherin, neural cell adhesion molecule, and retinoic acid receptor alpha. Moreover, theanine was ineffective in significantly affecting *Slc38a1* transcript expression in primary cultured neurons isolated from embryonic mouse neocortex, NSC34 motoneuronal cells, and KT5 astrocytic cells at a concentration range between 1 and 100 μM in vitro (our unpublished data). Theanine would selectively up-regulate Slc38a1 expression in primitive NSCs/NPCs, but not in daughter cells such as neurons and astrocytes. The reason for this selectivity for target molecule and cell phenotype, however, should be clarified in future studies.

### 3.2. Usefulness of Pluripotent P19 Cells as NPC Model

In order to evaluate the molecular mechanism underlying the upregulation by theanine of Slc38a1 expression, we have employed a cell line model featuring a phenotype similar to NPCs for artificial gene modifications. Mouse embryonal carcinoma P19 cell has a primitive cellular feature similar to the primitive ectoderm with a common ability to commit differentiation to a neural phenotype in the presence of retinoic acid [33,34]. As seen in the aforementioned NPCs from embryonic rodent brains, nestin-positive round spheres are formed during the in vitro culture with retinoic acid in the presence of growth factors. Subsequent culture without growth factors after dispersion leads to the appearance of cells immunoreactive for a neuronal marker and an astrocytic marker, in addition to the disappearance of nestin-positive cells. Exposure to theanine markedly increased the size of round spheres as well as MTT, reducing activity in undifferentiated P19 cells [35], with a concomitant increased number of cells immunoreactive for a neuronal marker and a decreased number of cells immunoreactive for an astrocytic marker after spontaneous differentiation. These findings allow us to use the embryonic carcinoma P19 cell line as a model of NPCs for elucidating the underlying mechanism as well as the functional significance of the upregulation by theanine of Slc38a1 expression.

### 3.3. Stimulation by Theanine of Slc38a1 Promoter in P19 Cells

One luciferase reporter analyzed using different elements from −959 bp to −768 bp upstream of Slc38a1 promoter [21]. Luciferase activity was more than doubled in P19 cells transfected with a full-length plasmid after sustained exposure to theanine. On deletion of plasmid analysis, however, the promoter region between −1626 and −768 bp upstream was found to be absolutely required for the upregulation by theanine of Slc38a1 expression in pluripotent P19 cells. In silico analysis revealed the presence of different elements responsive to particular transcription factors within the promotor region up to −3000 bp upstream of the *Slc38a1* gene [36]. These included activator protein-1 (AP1) and cyclic AMP responsive element-binding protein (CREB), which were both shown to be able to stimulate the promoter activity of the *Slc38a1* gene in C6 glioma cells [37]. In P19 cells transfected with the full-length promoter region of the *Slc38a1* gene, however, no marked simulation was induced for luciferase reporter activity even after the introduction of an expression vector of either AP1 or CREB [21]. In our hands, eightfold stimulation was only detected in Slc38a1 promoter activity in P19 cells with an expression vector of X-box binding protein-1 among 13 different expression vectors for intracellular signaling molecules well-known to date. Although theanine indeed up-regulates Slc38a1 expression through gene transcription for the promotion of embryonic neurogenesis, the exact molecular mechanism underlying the upregulation is still not clarified so far.

### 3.4. Establishment of Stable Transfectants of Slc38a1 in P19 Cells

The fact that theanine facilitated both embryonic neurogenesis and Slc38a1 expression in NPCs and P19 cells before differentiation gives rise to the idea that Slc38a1 may play a crucial role in molecular mechanisms underlying the promotion by theanine of embryonic neurogenesis in NSCs. In order to evaluate this possibility, we have established stable transfectants of Slc38a1 in pluripotent P19 cells [35]. Stable overexpression of Slc38a1 alone led to marked promotion of proliferation for self-renewal along with facilitated neuronal commitment even in the absence of theanine in undifferentiated P19 cells as expected. However, theanine failed to stimulate additively both proliferation and neuronal commitment activities in these stable Slc38a1 transfectants, in spite of marked promotion of both activities in P19 cells with an empty vector. The complete absence of the additivity between Slc38a1 overexpression and theanine exposure is favorable for a proposal that theanine promotes both proliferation and neuronal commitment through a molecular mechanism associated with the upregulation of Slc38a1 expression in primitive NSCs before differentiation in embryonic brains. The possibility that molecular structural similarity between theanine and GLN is at least in part responsible for the promoted neurogenesis in NPCs is thus conceivable.

## 4. Roles of GLN in the Brain

### 4.1. Replenishment of Amino Acid Neurotransmitter Pools

In addition to a role in energy production and protein synthesis as a general amino acid, GLN has been believed to replenish the neurotransmitter pool, such as synaptic vesicles, of Glu after the incorporation mediated by the GLNT isoform Slc38a1 and subsequent conversion to Glu by the catalytic action of glutaminase (GLNase) in glutamatergic neurons. A neuronal stimulus induces the exocytotic release of Glu from such synaptic vesicles, followed by removal from synaptic clefts by excitatory amino acid transporters for the conversion by GLN synthetase to GLN in adjacent astrocytes. Astrocytic GLN is exported to extracellular spaces across different GLNT isoforms, such as Slc38a3 and Slc38a5, for the entry mediated by Slc38a1 in neighboring neurons to replenish the glutamatergic neurotransmitter pool [38]. However, an inhibitor selective for Slc38a1 depleted both Glu and GABA neurotransmitter pools in guinea-pig cortical slices [39]. Oral administration of GLN at a high dose increased the endogenous levels of GABA and GLN, but not of Glu, in rat striatum on a determination by microdialysis [40]. In a recent report on mice devoid of Slc38a1 [41], furthermore, Slc38a1 was shown to play a key role to replenish the neurotransmitter pool of GABA in GABAergic neurons, along with a role in regulating normal cortical development and presynaptic inhibitory synaptic plasticity. The non-neuroactive amino acid GLN seems to participate in the replenishment of the neurotransmitter pools ready for prompt exocytotic release upon stimuli for Glu and GABA at individual synapses in the brain. Taking into consideration the universal role of both Glu and GABA as an extracellular signal mediator in organs other than the brain, we should re-evaluate the physiological and pathological significance of GLN in any cells expressing particular GLNT isoforms including Slc38a1, Slc38a3, and Slc38a5.

### 4.2. Modulation of Ammonia Homeostasis

In neurons, GLN is metabolized to Glu and ammonia by the catalytic action of phosphate-dependent GLNase [42] for the above-mentioned condensation at glutamatergic synaptic vesicles in the brain. Although GLNase is supposed to predominate exclusively in neurons for years [43], evidence is now accumulating for the co-localization of at least two GLNase isoforms with different molecular structures and regulatory profiles throughout the brain [38]. The L-type isoform is localized in both mitochondria and nuclei in neurons [44], while the K-type isoform is shown to be present in rat brain astrocytes on immunohistochemical analysis without the demonstration of validated functionality [45]. A positive correlation is seen between brain GLN levels and the severity of neurological symptoms in patients suffering from hyperammonemia resulted from impaired hepatic clearance of excessive ammonia to urea [38]. As both GLNase and Slc38a1 predominantly reside in neurons, at any rate, upregulation of Slc38a1 would lead to severe neuronal dysfunctions through a mechanism related to over-incorporation of extracellular GLN for the excess liberation mediated by GLNase of the neurotoxin ammonia in a particular situation. However, theanine is not a good substrate for this phosphate-dependent GLNase in the brain, but is metabolized by phosphate-independent GLNase responsible for the degradation of GLN to Glu and ethylamine in the kidney [46].

### 4.3. Pharmacological Profiles of [^3^H]GLN Uptake

Extracellular GLN was for the first time demonstrated to be accumulated in rat brain slices [47]. Among a variety of transporters capable of translocating GLN across plasma membranes, Slc38a1 isoform is thought to predominate in neurons with a high affinity for GLN in the brain [48,49,50]. [^3^H]Theanine binding was completely absent from rat brain synaptic membranes exhibiting high-affinity [^3^H]Glu binding sensitive to NMDA, while theanine was unable to affect sodium-dependent and high-affinity [^3^H]Glu uptake in rat brain synaptosomes [51]. However, both [^3^H]GLN and [^3^H]theanine were markedly accumulated in a temperature-dependent and saturable manner in rat brain synaptosomes [51]. [^3^H]GLN accumulation was inhibited by several amino acids structurally related to GLN, including theanine, in a concentration-dependent manner. [^3^H]Theanine accumulation was sensitive to the inhibition by GLN and relevant amino acids and *vice versa*, whereas theanine was not an inhibitor of [^3^H]Glu accumulation as described above. Moreover, Glu was a poor inhibitor of [^3^H]GLN and [^3^H]theanine uptake even at a high concentration. The close accumulation profile gives support to the idea that common GLNT isoforms are at least in part responsible for the translocation of both GLN and theanine across neuronal plasma membranes in the brain. On the basis of the aforementioned findings along with marked structural difference at the gamma carbon position, the possible interaction of theanine with either receptor or transporter for Glu is not conceivable so far.

### 4.4. Activation by GLN of mTOR Signaling

The mechanistic target of rapamycin (mTOR) is a serine/threonine protein kinase encoded by a gene located at chromosome 1p36.2 [52]. Intracellular GLN is supposed to modulate the activity of this kinase required for the harmonized integration of a variety of extracellular growth signals by nutrients through two distinct multiprotein complexes such as mTOR complex-1 (mTORC1) and mTOR complex-2 (mTORC2) toward the regulation of proliferation, migration, growth, autophagy, metabolism, and vitality in cells. Extracellular GLN is a prerequisite for activation of the mTOR signaling system in Jarkat cells [53], whereas intracellular GLN is one of the determinants crucial for the activation of mTORC1 signaling along with several GLN transporters in HeLa cells [54]. In particular, mTORC1 signaling is a downstream signal of extracellular essential amino acids (EAAs) [55]. Increased intracellular GLN levels lead to facilitation of the influx of extracellular EAAs in exchange for the efflux of intracellular GLN for the activation of mTOR1 signaling to the acceleration of cell growth and proliferation [54].

### 4.5. Significance of Selective Upregulation of Slc38a1 Expression

As mentioned above, theanine selectively up-regulated transcript expression of Slc38a1 among different trophic and adhesion factors capable of modulating primitive features in NPCs without affecting those in daughter cells in vitro. Taking into consideration the findings in stable Slc38a1 transfectants, intracellular GLN seems to play a key role in the promotion by theanine of the generation of new neurons through a mechanism associated with the aforementioned activation of intracellular mTORC1 signals in cultured NPCs. In fact, more than doubled intracellular GLN levels were detected with the concurrent promotion of both cell growth and neuronal commitment in stable Slc38a1 transfectants in P19 cells [35], in addition to increased phosphorylation of mTOR and downstream proteins [26]. The failure of theanine in stable Slc38a1 transfectants is suggestive of a similar common mechanism between sustained exposure to theanine and stable overexpression of Slc38a1 with regards to the promotion of proliferation and neuronal commitment in undifferentiated NPCs. In murine NPCs cultured with theanine [26], phosphorylation was drastically stimulated for mTOR and its downstream proteins. In stable Slc38a1 transfectants, similarly, marked phosphorylation was detected for mTOR, p70S6K, and S6 without alteration of the endogenous level of p70S6K. Theanine markedly accelerated the phosphorylation of mTOR and relevant downstream proteins in control mock transfectants as found in NPCs, without additionally stimulating the phosphorylation of these mTOR signaling proteins in stable Slc38a1 transfectants. Sustained exposure to theanine as well as stable Slc38a1 overexpression could commonly stimulate cell growth and neuronal commitment through a mechanism related to the activation of the mTORC1 signaling pathway in cultured NPCs in vitro. Indeed, the primitive profiles are reported to be under the delicate control by mTOR signaling toward the expression of particular basic helix-loop-helix (bHLH) transcription factors in P19 cells [56]. Activation of mTOR signaling is supposed to be responsible for proliferation, differentiation, migration, and maturation of neurons during neurogenesis [57,58].

### 4.6. Mechanisms Underlying Upregulation of Slc38a1 Expression

The exact molecular mechanism by which theanine up-regulates Slc38a1 transcript expression in NPCs is not fully clarified so far. One possible but hitherto unidentified possibility is that Slc38a1 is up-regulated through a mechanism compensatory to the sustained inhibition by theanine of the incorporation of extracellular GLN as seen with denervation supersensitivity in receptor pharmacology [59,60]. Intracellular signals mediated by cAMP and Ca^2+^ ions are often involved in mechanisms underlying the upregulation of a variety of membrane receptors after sustained inhibition by different antagonists. Although the gene encoding Slc38a1 really contains plural elements responsive to cAMP and Ca^2+^ signals at the upstream promoter regions responsible for the upregulation, artificial introduction of either AP1 or CREB is ineffective in activating the luciferase activity in P19 cells with the responsive full-length region of Slc38a1 promoter as mentioned above. Alternatively, it is still possible to speculate that theanine is incorporated across GLNT isoforms different from Slc38a1 to modulate intracellular mTOR signaling to neurogenesis. In our hands, however, theanine itself failed to activate the phosphorylation of mTOR and relevant downstream proteins in P19 cells (unpublished data). Furthermore, GLN was shown to be metabolized to α-ketoglutarate in the cytoplasm, which may then activate intracellular mTOR kinase signaling in collaboration with the EAA leucine [61]. The possible modulation by theanine of mTOR signaling through a mechanism irrelevant to Slc38a1 upregulation is not completely ruled out so far.

### 4.7. Conditional Knockout Mice Devoid of Slc38a1 from Neurons

In order to evaluate the physiological and pathological significance of Slc38a1 in the brain, we have at first made conditional knockout mice deficient of Slc38a1 from neurons expressing synapsin-I [62]. On immunohistochemical analysis using sections from the cerebral cortex of adult wildtype mice, Slc38a1 protein was co-localized with the neuronal marker protein neuronal nuclei, but neither with the astrocytic marker protein S100β nor with the microglial marker protein CD11b. In animals devoid of Slc38a1 from cells expressing synapsin-I, less damage was seen in coronal sections along with attenuated phosphorylation of mTORC1-relevant proteins after middle cerebral artery occlusion compared with sham-operated mice. In line with these findings in conditional knockout mice described above, the mTORC signal inhibitor rapamycin has previously shown to protect against ischemic damage through the facilitation of autophagy [63,64,65]. In neurons, Slc38a1 would thus increase the intracellular levels of GLN to activate mTOR signaling toward the exacerbation of ischemic damage via autophagy under a particular condition. In our previous in vitro culture studies using rat cortical astrocytes [37] and astrocytic C6 glioma cells [66], artificial overexpression of Slc38a1 invariably led to exacerbated vulnerability to oxidative stress by hydrogen peroxide. We need to clarify the reason for the differential expression profiles between transcript and protein for Slc38a1 in astrocytic cells on different analyses. Nevertheless, temporary expression of a variety of genes unique to primitive cells could account for a particularly different role of intracellular mTOR signals in mechanisms underlying the regulation of both proliferation and neuronal commitment in undifferentiated NSCs. The final conclusion should await the future establishment and investigation of conditional knockout mice deficient of Slc38a1 from NPCs/NSCs.

## 5. Theanine for Human Brain Wellness

Oral ingestion of theanine has been shown to be beneficial for brain wellness in healthy humans as well as patients suffering from particular neurodegenerative diseases and neuropsychiatric disorders for years. In healthy human subjects, oral ingestion of theanine led to protection against different responses to psychological and physiological stressors [67]. Oral intake of theanine was more effective in alleviating the elevations of both anxiety and blood pressure than caffeine in human adults with stressful tasks [68]. In an open clinical study on patients with major depressive disorder, similarly, several improvements were seen in the therapeutic efficacy of the current medication in terms of depressive symptoms, anxiety, sleep disturbance, and cognitive impairments after daily oral ingestion of theanine at a dose of 250 mg/day for eight weeks [69]. In boys previously diagnosed with attention deficit hyperactivity disorder (ADHD) at ages of eight to 12 years old, the quality of sleep was markedly improved after daily oral intake of theanine at a dose of 400 mg/day for six weeks in a randomized, double-blinded, and placebo-controlled study [70]. In patients with ADHD-relevant sleep disturbance, theanine was reported to improve sleep efficacy rather than sleep latency [71]. Oral administration of theanine at 200 mg before sleep alleviated the quality of sleep in a manner related to anxiolysis and relaxation through increased alpha brain wave in humans [72], while theanine was without any remarkable adverse side effects even after oral ingestion at 2 g/kg body weight/day [72]. The latter fact is highly suggestive of the qualified safety of oral intake of theanine even at an extraordinarily high dose in humans.

In patients with chronic schizophrenia and schizoaffective disorders, moreover, ongoing antipsychotic effectiveness was facilitated by oral intake of theanine given at a dose of 400 mg/day for eight weeks in a fashion related to reduced anxiety [73]. The beneficial effectiveness was positively correlated with serum levels of brain-derived neurotrophic factor in schizophrenic and schizoaffective patients orally given theanine for eight weeks [74]. Therapeutic benefits were also predicted for theanine in other psychiatric disorders, including anxiety, panic, and obsessive-compulsive and bipolar disorders [75]. Although a direct positive correlation between experimental promotion of adult neurogenesis and clinical therapeutic benefits is not fully clarified to date as mentioned above, the green tea amino acid theanine seems to alleviate a variety of symptoms and syndromes associated with dysfunctions of harmonization of the intact neuronal network toward brain wellness with guaranteed safety in young to adult humans. Sustained oral intake of theanine could be beneficial for the prophylaxis and treatment of different neurodegenerative and neuropsychiatric disorders through a mechanism related to promoted birth of new neurons from NSCs in embryonic, postnatal, juvenile, adolescent, adult, and/or senile brains in a particular situation.

Theanine has been believed to easily gain access to the brain across the blood–brain barrier through absorption from the intestinal mucosa to the circulating blood after oral ingestion [76,77]. In contrast, neuroprotective polyphenols such as catechins are most abundant in green tea with a poor bioavailability for the brain after oral consumption [78,79,80]. Although polymeric nanoparticle-based delivery systems could be useful for the efficient delivery of neuroprotective catechins to the brain [81], such artificial modifications would often lead to severe impairment, dysfunction, and degeneration in a variety of eukaryotic cells in a particular situation. At any rate, we need to establish and develop validated nutraceutical sciences as quickly as possible as done with pharmaceutical sciences for human healthcare [82].

## 6. Conclusions

**Figure 1:**
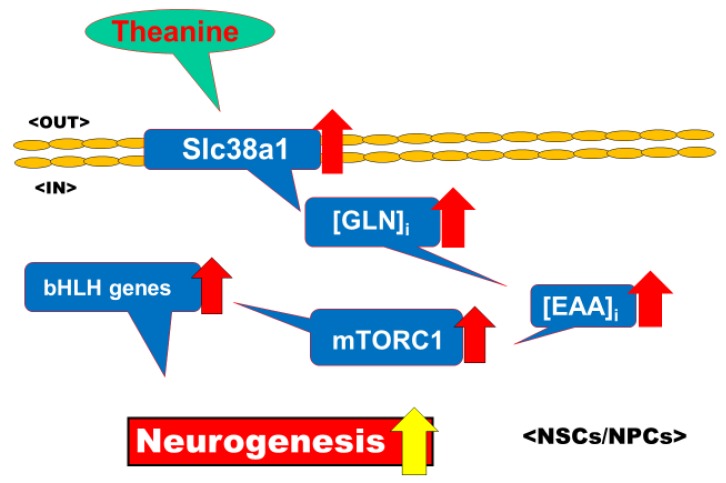
Proposed signaling from theanine to neurogenesis. Sustained exposure to theanine selectively up-regulates the expression of an isoform of glutamine transporter (GLNT), Slc38a1, among different adherent and trophic molecules endowed to modulate the properties of neural stem cells (NSCs) toward an increase in the intracellular glutamine (GLN) level in primitive NSCs/neural progenitor cells (NPCs) only, but not in daughter cells such as neurons and astrocytes. In exchange of intracellular GLN, the incorporation of extracellular essential amino acids (EAAs) is facilitated to activate mTORC1 phosphorylation signaling to upregulation of several basic helix-loop-helix (bHLH) transcription factors capable of promoting neurogenesis in NSCs/NPCs. In neurons and astrocytes, by contrast, theanine is unable to up-regulate the expression of Slc38a1, which is absolutely required for triggering the proposed signaling cascade.

Sustained exposure to theanine would lead to selective upregulation of Slc38a1 expression toward the increased intracellular GLN level, followed by facilitated incorporation of extracellular EAAs in exchange of intracellular GLN in primitive NSCs/NPCs only, but not in daughter cells such as neurons and astrocytes. Resultant increased EAA levels could activate mTORC1 phosphorylation signaling to upregulation of several bHLH transcription factors capable of promoting neurogenesis in NSCs/NPCs, as summarized in Figure 1. In neurons, by contrast, theanine should suppress the replenishment of neurotransmitter pools of inhibitory GABA as well as excitatory Glu through a mechanism relevant to inhibition of the incorporation mediated by different isoforms of GLNT of extracellular GLN. The latter action could at least in part account for the improvement of sleep disturbance after acute theanine intake in humans, while the former action would be related to the usefulness for the preservation of brain wellness after chronic theanine ingestion in a particular situation. Both actions seem to be commonly mediated by the inhibition of GLN transport by theanine. The exact molecular mechanism underlying the selective upregulation of Slc38a1 expression by theanine in primitive NPCs/NSCs only, however, still remains to be elucidated in future studies.

## Abbreviations

ADHD: attention deficit hyperactivity disorder; AP1, activator protein-1; bHLH, basic helix-loop-helix; BrdU, 5-bromo-2′-deoxyuridine; CREB, cyclic AMP responsive element-binding protein; EAA, essential amino acids; GABA, γ-aminobutyric acid; GFP, green fluorescent protein; GLN, glutamine; GLNase; glutaminase; GLNT, glutamine transporter; Glu, glutamic acid; mTOR, mechanistic target of rapamycin; mTORC, mechanistic target of rapamycin complex; MTT, 3-(4,5-dimethyl-2-thiazolyl)-2,5-diphenyl-2H-tetrazolium bromide; NMDA, N-methyl-D-aspartic acid; NPCs, neural progenitor cells; NSCs, neural stem cells; Slc38a1, solute carrier 38a1; SNAT1, sodium-dependent neutral amino acid transporter-1; SGZ, subgranular zone; SVZ, subventricular zone.
